# Investigating the anti-tumoral effect of curcumin on the mice in which Ehrlich ascites and solid tumor is created

**DOI:** 10.22038/ijbms.2019.33623.8019

**Published:** 2019-04

**Authors:** Seher Yılmaz, Harun Ülger, Tolga Ertekin, Arzu Hanım Yay, Mehtap Nisari, Şerife Alpa, Niyazi Acer

**Affiliations:** 1 Department of Anatomy, Yozgat Bozok University Faculty of Medicine, Yozgat, Turkey; 2 Department of Anatomy, Erciyes University Faculty of Medicine, Kayseri, Turkey; 3 Department of Anatomy, Kocatepe University Faculty of Medicine, Afyon, Turkey; 4 Department of Histology, Embriology, Erciyes University Faculty of Medicine, Kayseri, Turkey; 5 Department of Anatomy, Karatay University Faculty of Medicine, Konya,Turkey

**Keywords:** Animal, Apoptosis, Cell, Curcumin, Factor VIII, Injection, Mice

## Section Title

### Objective(s):

In this study, the effects of different doses of curcumin application on Ehrlich ascites tumor (EAT) created in the mice of BALB/c type were investigated.

### Materials and Methods:

Curcumin extracts can have hindering effect on tumor volume, vascular density, EAT cells around the tissues, and can support apoptosis. EAT cells (1x10^6^) received from stock animals were injected intraperitoneally (IP) and subcutaneously (SC) to the animals. Then, curcumin was administered IP. Doses of 25 mg/kg IP and 50 mg/kg were administered over 10 days to the animals in the treatment groups in which ascites tumor was induced. The same doses were administered over 15 days in the treatment groups in which solid tumor was induced.

### Results:

Histopathological examination in ascites tumor groups revealed that number of EAT cells at surrounding tissues was smaller in the group received 50 mg/kg curcumin when compared to tumor control group (*P<*0.05). The lowest increase in tumor volume was observed in the group received 25 mg/kg curcumin when compared to tumor control group (*P<*0.05). 

### Conclusion:

It was demonstrated once again in our study that curcumin had an anti-tumoral effect on both the development of ascites tumor created through EAT cells and the development of solid tumor.

## Introduction

An average of 10 million diagnoses of cancer are established every year; and each year, more than 6 million people die of cancer. Today, there are more than 22 million cancer patients all around the world (1-4). In the treatment of cancer, the complementary medicine is also benefited from in addition to chemotherapy and radiotherapy. Herbal extracts are benefited from in complementary medicine, the usage of which is increasing with each passing day and (5). One of these herbal extracts is the curcumin. A member of Zingiberaceae family, Curcuma Longa is a perennial plant, the native land of which is the Southern Asia. Its original supplier is India; yet, it is also grown in Bangladesh, China, Indonesia, Caribbean Islands and in some of the countries of South America as well. Known as turmeric powder, Curcuma longa is used in the production of curry powder for meals due to its aroma and yellow colour. Separately, it is also produced as tea in various regions of Japan (6, 7). There are several articles in the literature which put forward the antineoplastic mechanism of curcumin. It is known that curcumin increases the mortality rate of some types of cancer cells and inhibits the division of tumour cells (8). The antioxidant and anti-tumoral effects of herbal extracts have been experimented on many cancer models; and one of them is Ehrlich Ascites Tumour (EAT) model. EAT emerged for the first time as a spontaneous breast adenocarcinoma in a female mouse, and the tumour pieces were transplanted subcutaneously from mouse to mouse, after which they were turned into experimental tumours. Afterwards, the growing form of the tumour in liquid form in the peritoneum of mice was obtained, and since ascitic fluid besides cells also occurred in the peritoneum, the tumour was called Ehrlich Ascites Tumour (9, 10).

The objective of this study is to investigate the anti-tumoral effect of curcumin on the models of EAT ascites and solid tumour created experimentally in the mice of Balb/C type. 

## Materials and Methods

**The Type, the Number, and the Distribution of Experimental Animals Used in the Research**

The present study, which was performed on experimental animals, was carried out in accordance with the decision dated Feb/12/2014 and numbered 14/029, which was received from the Local Ethical Committee of the Animal Testing Department of Erciyes University. During the study period, the male mice of Balb/C type, which were 8-10-week-old and weighed 25-30 gr, were used. While 8 groups were formed in a way that there would be 10 mice in each group, 4 animals were used to form a group of stock animals apart from the groups in question. Throughout the study period, the mice were kept at a constant temperature of 21ºC in the privately-prepared and automatically-climatized rooms in which there was a 12-hr-daylight and 12-hr-night-time cycle. During the study, stock mice were arranged in the first place so as to obtain a sufficient amount of EAT cells prior to the formation of the groups. The EAT cells received from the stock mice were used for *in vitro* cell culture along with *in vivo* liquid and solid tumour formation. 

**Dissolution and Sterilization of the Curcumin Extract**

Curcumin was supplied as powder from Sigma Aldrich. On the day of the experiment, the curcumin extract was dissolved in different volumes via dimethyl sulfoxide (DMSO) and Phosphate Buffer Saline (PBS) buffers in a way that it would provide the desired concentrations for each experimental group. 

**Formation of the stock mice**

The EAT cells that were kept at -80^o^C were used in forming the stock animals. The stock cells were thawed at room temperature and was administered to stock animals as 0.1 ml in intraperitoneal way at the joint of the left rear leg and the stomach area. It was expected that ascites tumour would occur in the stock animal within 6-7 days. The 1x10^6^ EAT cells in the ascites fluid drawn with the help of an injector from the stock animal were administered to the mice intraperitoneally in 0.1 ml PBS to form a liquid tumour; and it was administered to the neck area in a subcutaneous manner to form solid tumour. The EAT cells in 1 ml of the fluid taken from the intraperitoneal space of the stock animal was determined according to the following procedure.

**Forming the Experimental Groups**

**Ascites Tumour Groups**

**Group 1: Negative Control Group (n=10): **No cancer was created, and the animals in question were fed on a normal diet for 10 days. 0.1 ml of PBS was administered through the IP. way for 10 days. 

**Group 2: Positive Control Group (n=10): **The mice included in this group were administered 1x10^6^ EAT cell through the IP way on the 1^st^ day. Starting from the 1^st^ day onwards, 0.1ml of PBS was injected to the mice through the IP way for 10 days. 

**Group 3: Treatment Group (25 mg-curcumin)** **(n=10): ** The mice included in this group were administered 1x10^6^ EAT cells through the IP. way on the 1^st^ day. Starting from the 1^st^ day onwards, 25mg/kg/day curcumin was injected to the mice through the IP way for 10 days. 

**Group 4: Treatment Group (50 mg-curcumin) (n=10): **The mice included in this group were administered with 1x10^6^ EAT cells through the IP way on the 1^st^ day. Starting from the 1^st^ day onwards, 50mg/kg/day curcumin was injected to the mice through the IP way for 10 days. 

Starting from the day on which the experiment was started until the last day of the experiment, the weights of the animals were measured. In the ascites tumour experiment groups on the 10^th^ day, in the solid tumour experiments groups on the 15^th^ day, all of the mice in these groups were sacrificed; and solid tumour groups were sacrificed on the same day and the tumour size was observed and sacrificed with the ketamine xylazine. The ascites group mice underwent general anaesthesia with ketamine doses that were calculated per mice (100mg/kg) and xylazine (10mg/kg); and the anticarcinogenic effect of curcumin was evaluated by performing a cell count. 

**Solid Tumour Groups**

**Group 1: Negative Control Group (n=10): **No cancer was created. The animals were fed on a normal diet for 15 days; and 0.1 ml of PBS was administered through the SC way for 15 days. 

**Group 2: Positive Control Group (n=10): **On the 1^st^ day, 0.1 ml of ascitic fluid containing 1x10^6^ EAT cells was injected through the SC way into the back-neck areas of the mice included in this group. Starting from the 1^st^ day onwards, 0.1 ml of PBS was injected into the mice through the SC way for 15 days. 

**Group 3: Treatment Group (25 mg-Curcumin)** **(n=10):** On the 1^st^ day, 0.1 ml of ascitic fluid containing1x10^6^ EAT cells was injected through the SC way into the back-neck areas of the mice included in this group. Starting from the 1^st^ day onwards, 25 mg/kg/day curcumin was injected into the mice through the SC way for 15 days. 

**Group 4: Treatment Group (50 mg-Curcumin)** **(n=10): **On the 1^st^ day, 0.1 ml of ascitic fluid containing1x10^6^ EAT cells was injected through the SC way into the back-neck areas of the mice included in this group. Starting from the 1^st^ day onwards, 50 mg/kg/day curcumin was injected into the mice through the SC way for 15 days. The solid tumour volumes that developed in the animals throughout 15 days were measured through an electronic compass (*Tumour Volume (mm*^3^*) = Width²XLengthX0,52*) (11). In the wake of the experiment, the tumour tissue samples taken from the animals were fixed through 10% of formaldehyde solution for histopathological examinations. 

**Immunohistochemical Practices:**

**Apoptosis Tunel Staining Method**

As for the tumour tissue sections pertaining to the control and experimental groups (10 mice in each group), the most sensitive and the most rapid method, TUNEL (Terminal deoxynucleotidyl transferase-mediated dUTP Nick end Labelling) labelling method, was used in the* in situ* detection of the DNA spiral fractures in the apoptotic cells. In brief, 5μm-sections that were put in the laminae covered with Poly-L-Lysine were deparaffinized in xylene, and then they were exposed to the decreasing alcohol series and were then incubated in PBS (Phosphate Buffer Saline) for 5 min at room temperature. After the sections that were incubated with proteinase K for 15 min were washed in distilled water, they were treated with 30% of H_2_O_2_ so as to suppress the endogenous peroxidase activity. For the succeeding stages, an immunofluorescent staining was performed in accordance with the staining method found within the kit (Apoptaq In Situ Cell Death Detection Kit, Millipore, S7110) as recommended by the manufacturing company. For this process, the sections that were washed up with PBS were incubated along with TdT enzyme at 37°C in a dark and humid environment for 60 min. The sections washed up with PBS once again began to react with anti-digoxigenin conjugate at room temperature. All the incubation stages were fulfilled in a humid environment. In order to obtain images, the slides were placed under the immunofluorescent microscope (Olympus BX 51).

The apoptotic cells were determined in the images obtained in the wake of Fluorescent Isothiocyanate (FITCH) and 4',6-diamidino-2-phenylindole (DAPI).

**Factor VIII Staining**

Factor VIII Staining is one of the methods used in determining the increasing angiogenesis in the tumour tissue. For this reason, we used this method in the present study of ours. Avidin-Biotin-Peroxidase method was performed on the solid tumour tissues obtained from mice to determine Factor VIII expression. In our study, 10 mice in each group and the tumour tissue sections put into the laminae with Polylysine were placed into the cassettes/trays and were incubated at 60˚C in the incubator for one night. Later on, they were subjected to xylene and then to the alcohol series at decreasing degrees, after which they were rehydrated. They were washed up with distilled water for three times and for 2 min each. Afterwards, for the retrieval of antigen, 10% citrate buffer was exposed to 5-min-heat at 600 W in a microwave oven, after which it was left for cooling for 10 min at room temperature. The sections that were washed up with PBS were treated with 3% of hydrogen peroxide (H_2_O_2_) for 12 min in order to prevent the endogenous peroxidase activity. They were then washed up with PBS once again. A staining kit (Thermo Scientific) was used for the succeeding stages. After the washing process, which was repeated, it was treated with peroxidase substrate, which was in the kit that had diaminobenzidine (DAB) (Thermo Scientific) feature, for 1.5 min in order to make the immune-reactivity to become visible. The sections, which were contrast-stained with gill haematoxylin, were washed with distilled water several times. Finally, the sections whose water was removed with remaining alcohol series, and which were passed through xylene, were closed with closure solution (Entellan®, Merck), and were examined in Olympus BX51 microscope (Olympus BX51, Tokyo, Japan).

***In vitro*** **Experimental Group**

The effects of 10 µg/ml, 20 µg/ml and 30 µg/ml-doses of curcumin on EAT cells were examined for the cell culture. Curcumin was dissolved within 10% of DMSO and PBS. The culture medium was prepared along with 20% Foetal Bovine Serum, 80% Dulbecco Middle Eagle Medium and 1ml-penicillin/streptomycin: 10,000 unit/ml penicillin, 10 mg/ml streptomycin. The plates with 96 sections were divided into 4 groups as tumour control group, 10 µg/ml, 20 µg/ml and 30 µg/ml curcumin treatment groups in the way that there would be 24 wells in each group; and 104.000 EAT cells were cultured into each well. Then, the effects of different doses of curcumin were evaluated by performing vital and dead cell counts 3 and 24 hr later.

**Cell count**

The EAT cells that were in the suspension form in the medium in each well were laced in the Eppendorf Tube; and 100 µl Trypan Blue was added by using a pipet. The Thoma slide was placed on a smooth surface and the lamellae was covered on the counting area frame to which the lamellae would be glued; and 50 µl cell solution was pipeted from the middle line to the counting area by placing the edge of the pipet to the point where the slide and the lamellae joins in the counting area which is in the middle of the channels placed on both sides of the Thoma slide. Then the Thoma Slide was placed on the plate of the microscope. While waiting for 15-20 sec, the objective of the microscope was adjusted to 40X and the counting area was found. The cells were counted one-by-one and the number of them were noted. 

**Statistical Analysis**

Histogram and *q-q* plots were examined, and the Shapiro-Wilk’s test was applied to assess the data normality. The Levene test was used to test the variance homogeneity. To compare the difference among groups, either one-way analysis of variance (ANOVA) or Kruskal Wallis test were applied for continuous variables. One-way analysis of variance was used in the comparison between groups. The Tukey test was used in multicomponent comparisons. Dunn-Bonferroni or Tukey tests were used for multiple comparisons. The Kruskal Wallis analysis was used in the intergroup comparisons. Dunn-Bonferroni test was used in multiple comparisons. For Factor VIII, and Apoptosis between the groups, Kruskal Wallis and One-Way Anova was used. For dose comparison in *in vitro* experimental groups, the Kruskal Wallis test was applied. The analyses were conducted using IBM SPSS Statistics **(Statistical Package for the Social Sciences) 22 program.** A *P-* value less than 5% was considered as statistically significant.

## Results

**Body Weight Changes in Ascites Tumour Groups throughout the Experiment**

When the data regarding the daily body weights of the animals in the groups throughout the experimental period (10 days) were examined, it was observed that there was an increase in the body weights of the tumour control group and curcumin treatment groups.

**Figure 1:**
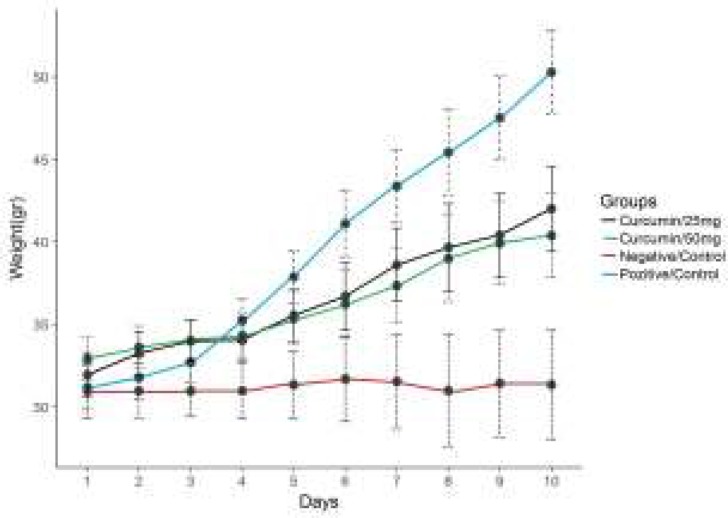
The average weight changes in the control and experimental groups (ascites tumor).

It was determined that there was more increase in the tumour control group than in the treatment groups. While the animal weights on the final day proved to be 50.3 gr in the tumour control group, the weights in 25 mg/kg-curcumin group belonging to the treatment groups proved to be 42.02 g, whereas the weights in the group to which curcumin was applied were recorded as 40.4 g (*P*<0.05) (Figure 1). 

Compared with treatment groups, tumor control group*P*<0.05

**Histopathological Results**

**Figure 2:**
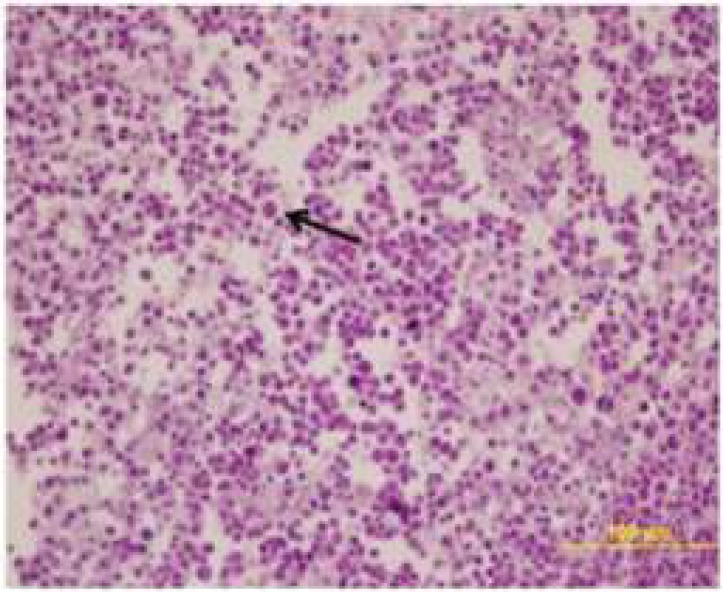
The histopathological image of EAT cells (Arrow = indicates to the EAT cells of different sizes) (H&E 40X)

**Figure 3:**
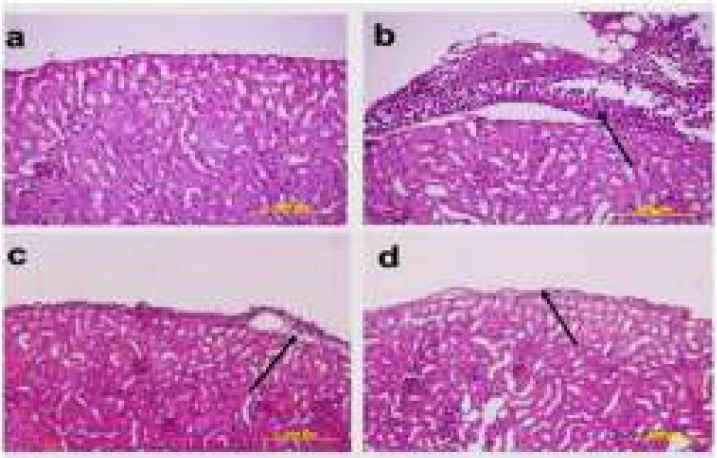
The histopathological findings in the kidney tissues pertaining to the healthy control group, treatment group and the tumour-application groups. **a) **Healthy control group **b) **Tumour control group **c)** The group to which tumour and 25mg/kg curcumin were applied **d) **The group to which tumour and 50mg/kg curcumin were applied (H&E, 20X)

**Figure 4:**
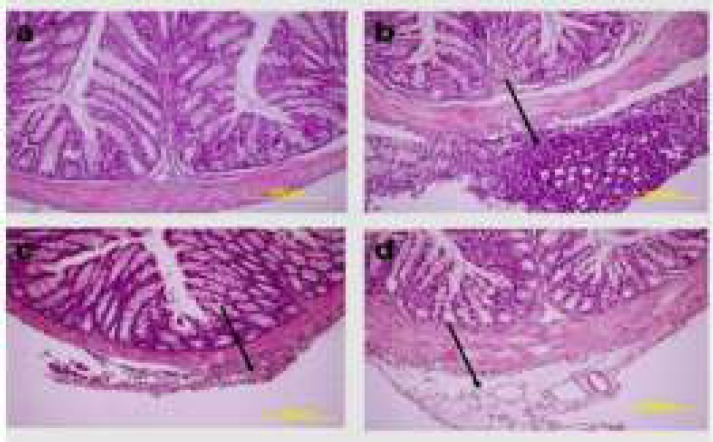
The histopathological findings received from the middle line of the large bowel tissue pertaining to the healthy control and treatment groups. **a) **Healthy control group **b) **Tumour control group **c)** The group to which tumour and 25mg/kg curcumin were applied **d) **The group to which tumour and 50mg/kg curcumin were applied (H&E, 20X)

**Figure 5:**
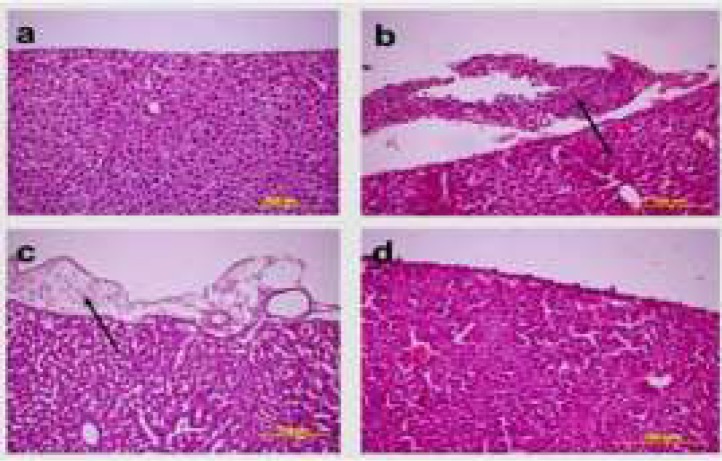
The (histopathological) findings of the liver tissue pertaining to the tumour control and treatment groups. **a) **Healthy control group **b) **Tumour control group **c)** The group to which tumour and 25mg/kg curcumin were applied **d) **The group to which tumour and 50mg/kg curcumin were applied (H&E, 20X)

It was determined that while the EAT cells in the kidney, liver and spleen tissues belonging to the healthy tumour control and treatment groups were invasive towards the connective tissue capsule, they were invasive towards the tunica serosa layer in stomach, small bowel and large bowel tissues; and the cells in question were ascertained to have bulky hyperchromatic nucleus, eosinophilic cytoplasm and different morphological characteristics. While EAT cells showed an intensive clustering in the tumour control group, they were observed individually around the connective tissue capsule in the treatment groups (Figures 2, 3, 4, 5).

**Body Weight Changes in Solid Tumour Groups throughout the Experiment**

**Figure 6:**
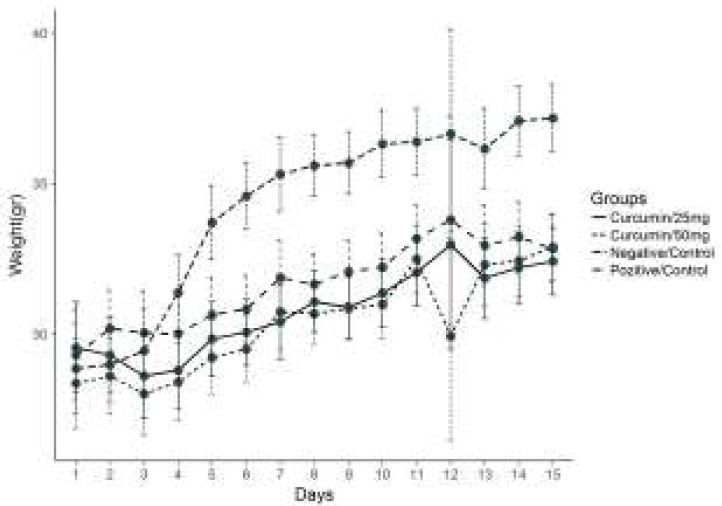
The average weight changes in the control and experimental groups(solid tumor) Compared with treatment groups, tumor control group *P*<0.05

Throughout the 15-day-experimental period, the mean body weights of the animals in the control and treatment groups were measured, and on the final day of the experiment, the weight in the tumour control group was 37.17 g, whereas it proved to be 33.01 g in the group administered with 25 mg/kg curcumin, and it was recorded as 32.98 g in the group administered with 50 mg/kg curcumin. The highest increase rate was observed in the tumour control group (*P<*0.05) (Figure 6). 

**Tumour-Volume Changes in Solid Tumour Groups throughout the Experiment**

The tumour size in the control and treatment groups was recorded by starting from the day on which the tumour volume could be measured from the surface of the skin. While the measurements were started to be performed in the tumour control group by starting from the 7^th^ day onwards, these measurements could be performed by starting from the 8^th^ day onwards in the treatment groups. The tumour volumes on the final day of the experiment were measured as 4603.99 mm^3^ in the tumour control group, 1179.56 mm^3^ in 25mg/kg curcumin group, and as 2059.12 mm^3 ^in 50 mg/kg curcumin group. The overall tumour sizes in all the animals were compared volumetrically among themselves, and when the treatment groups were compared with the tumour control group, the tumour-volume increase was found to be statistically significant (*P*<0.05). The findings pertaining to this comparison are given in (Table 1).

**Table 1:** Comparison of the solid-tumour volumes between the groups according to days

**Day**	**Tumor Control Group** ** (n=10)** **Median ( %25 – 75 )**	**Treatment Group 25mg/kg Curcumin (n= 10)** **Median ( %25 – 75)**	**Treatment Group 50mg/kg Curcumin (n= 10)** **Median ( %25 – 75 )**	***P***
**7**	60.42(49.29–110.71)	-	-	-
**8**	212.13(52.11–376.16)	72.54(19.02-354.76	159.98(39.85-195.49)	0.614
**9**	384.57(253.49-762.10)	138.07(77.79-263.49)	309.62(142.49-482.17)	0.118
**10**	1127.99(345.56-2156.91)	388.91(282.76-415.76)*	604.96(195.70-875.25)*	0.011
**11**	1763.52(514.33–2574.21)	478.30(335.68-677.29)*	1379.30(497.12-2451.48)*	0.008
**12**	1746.88 (494.36–3161.91)	606.99(341.79-773.41)*	1162.43(462.50-2303.32)*	0.008
**13**	2728.83(1363.49–3881.40)	810.26(477.03-1323.69)*	1857.10(639.81-2447.20)*	0.008
**14**	4086.90(2093.69–8049.06)	969.56(579.21-1669.80)*	2112.84(859.81-2864.16)*	0.006
**15**	4603.99(3196.06–8049.06)	1179.56(607.81-1910.22)*	2059.12(855.16-2059.12)*	0.006

Values ​​measured in mm^3^. Compared with tumor control group

* *P*<0.05 .

**Results of Angiogenesis**

**Figure 7:**
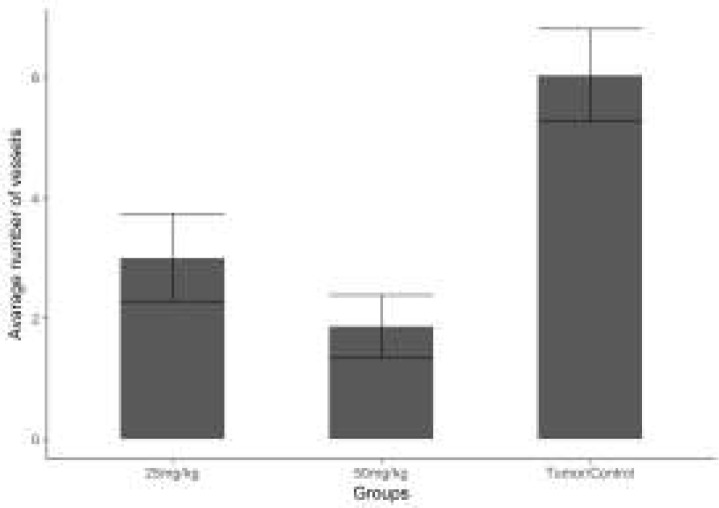
Comparison of the vascular density pertaining to Factor VIII between the experimental groups 25mg/kg and 50 mg/kg curcumin. Coordinate plane (0-6) is that the avarage number of vessels (Bar graphic). Compared with tumor control group *P<0.05

**Figure 8:**
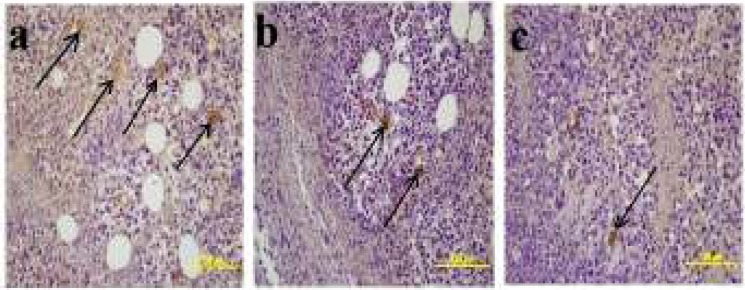
Factor VIII immunohistochemical staining. **a)** Negative control group **b) **Tumour control group **c)**Treatment group with 25mg/kg curcumin **d) **Treatment group with 50mg/kg curcumin- Factor VIII expression in the vascular endothelium (arrow = Factor VIII positive capillary)

The mean vascular density determined through Factor VIII in the sections obtained from the tissues of the groups are shown in (Figure 7). Through the immunohistochemical staining performed for Factor VIII, a positive staining was observed in the vascular endothelial cells in the sections belonging to the control and treatment groups (Figure 8). The necrotic regions of the tumour tissue were not included in the evaluation. 

When the vascular density between the groups was examined, the mean vascular density in the control group was calculated as 6.033, whereas in the treatment groups, it was calculated as 3.000 in the group to which 25mg/kg curcumin was applied and as 1.866 in the group to which 50mg/kg curcumin was applied. When the treatment groups were compared with the control group in terms of the density of Factor VIII expression, a statistically significant difference was determined between them (*P*<0.05).

**Apoptotic Results**

**Figure 9:**
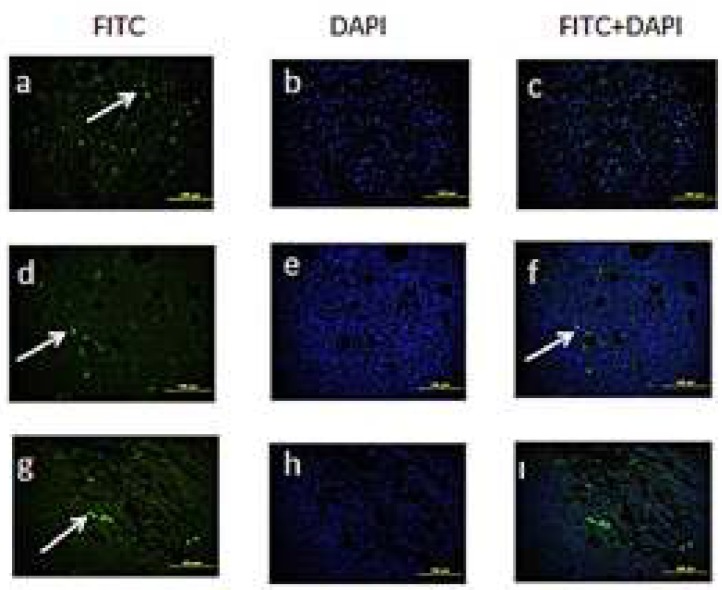
Apoptotic cells belonging to the experimental groups. **a-c) **Apoptotic cells belonging to the control group** d-f)** Apoptotic cells belonging to the treatment group (25mg/kg curcumin) **g-ı)** Apoptotic cells belonging to the treatment group (50mg/kg curcumin). Arrow = Apoptotic cells

**Figure 10:**
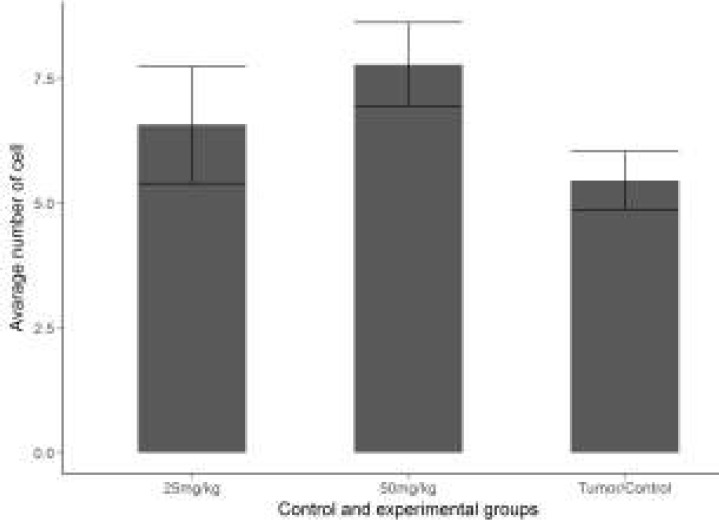
Comparison of the number of apoptotic cells between the tumor control and experimental groups 25mg/kg and 50 mg/kg curcumin groups. Coordinate plane (0-7.5) is that the avarage number of cell. (Bar graphic) Compared with tumor control group *P<0.05

It was observed in the solid-tumour tissue sections obtained from the experimental groups that the apoptotic cells had spread to the tumour tissue in general (Figure 9). The number of apoptotic cells is shown in Figure 10. According to the obtained results, a prominent increase was observed in the number of apoptotic cells in both of the treatment groups. We observed more apoptotic cells in the group to which we had administered high doses of curcumin when compared with the tumour control and other treatment groups. When the treatment groups were compared with the control group, the difference between them was determined to be statistically significant (*P*<0.05).

**The** ***in-vitro*** **effect of the curcumin extract applied to EAT cells**

**Figure 11:**
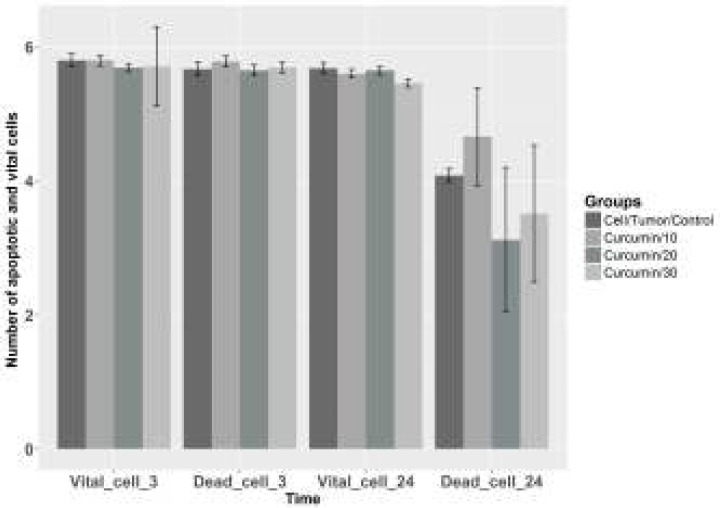
The avarage value of vital and dead cells calculated in the *in vitro* experimental group. Coordinate plane (0-6) is that the avarage number of cell. Compared with cell tumor control group *P<0.05

The effects of different doses of curcumin (10µg/ml, 20µg/ml and 30µg/ml) added onto the EAT cells on the number of live and dead cells in the wake of 3- and 24-hr-incubation periods were evaluated. In the counting processes performed after the two separate 3-hr- and 24-hr-incubation periods, the highest number of dead cells were determined to be in the group administered with 10µg/ml curcumin, and the numerical data obtained are given as the mean number of cells and as standard deviation in Figure 11. 

## Discussion

Cancer is a disease that mostly results in mortality as the result of uncontrolled cell growth and their rapid spread around the peripheral tissues. It is thought in the treatments of cancer that the combined application of surgery, chemotherapy and radiotherapy on patients extends the survival period of the patient (12-14). Some plants/herbs have been beneficial in the treatment of cancer, which has been shown in previously conducted studies. One of these herbs is the curcumin, which is found in the turmeric plant (15-17).

Yu-Wen *et al*. (18) investigated the preventive effect of curcumin (50mg/kg/day) on a case with acute pancreatitis that was experimentally created in mice; and after the experiment, it was reported that the damage in the pancreas had diminished, while the serum amylase level had increased. Ueki *et al*. (19) investigated the therapeutic effect of curcumin on the mice with renal inflammation which were agitated/stimulated by cisplatin; and it was stated that the serum TNF concentration in the group administered with curcumin (100mg/kg) along with cisplatin had decreased by 30%, whereas the serum TNF level had decreased by 10% in the group administered with only curcumin (50mg/kg), and that there was recovery in the renal dysfunctions as well. 

Bhaumik *et al*. (20) investigated the effects of curcumin on AK-5 tumour cells; and reported that there was a visible swelling in the peritoneal regions in the control group and that 90% mortality had occurred in the experimental group at the end of the 10^th^ day. As for the group administered with curcumin, on the other hand, it was reported that the rate of peritoneal swelling was determined as 80% and the animals within this group survived until after the 40^th^ day. 

Limtrakul *et al*. (21) investigated the repressive characteristic of curcumin on skin carcinogenesis in mice and applied 115 mg/kg/day-curcumin to the mice orally for 3 months. At the end of the experiment, they stated in their study that the curcumin dose had not reached toxic values in the blood serum level and that it induced apoptosis in the cancer cells that occurred in the mice. 

In our study we conducted, the highest increase in animal weights in the groups to which IP EAT tumour cell was administered was seen in the tumour control group, whereas the increase in weight in the treatment groups was determined to be less. In the wake of the histopathological examination; in the tumour group, invasive EAT cell groups with bulky hyperchromatic nucleus and eosinophilic cytoplasm in different forms were observed around the kidney, liver, spleen, stomach, and small and large bowel tissues taken from the tumour-positive and treatment groups. On the other hand, in the group administered with a high dose of curcumin along with EAT, the EAT cells around the tissue were not as dense as they were in the tumour control group. The tissue structures in this group exhibited normal histological characteristics. It was reported in the literature that when 1x10^6^ EAT cell suspension was injected subcutaneously into the host mouse, a solid tumour that could be measured at the end of a one-week-period had occurred. Armutak *et al*. (22) examined the effects of curcumin on apoptosis in the EAT solid tumour model; and applied 100 mg/kg curcumin to the animals in an IP way. While no difference was recorded in the tumour sizes of the experimental groups in their study, it was emphasized that 100 mg/kg of curcumin had increased apoptosis. 

In our study, we observed that the tumour size was on measurable dimensions in the control group by starting from the 7^th^ day onwards, and it was also measurable in the treatment groups by starting from the 8^th^ day onwards. We determined a decrease in the tumour volume in the group administered with a lower dose. The apoptosis-inducing effect of curcumin was studied on various cancer cell lines by researchers. 

Choudri *et al*. (23) reported that apoptosis had increased on the lung cancer cell lines after 24-hr and 48-hr-curcumin administration (10 µM and 25 µM) (24). Somasundaram *et al*. (24), on the other hand, reported that curcumin had increased apoptosis in human breast cancer cell, whereas Bhaumik *et al*. (25) reported that curcumin caused an increase in apoptosis on AK5 cell line, and Weir *et al*. stated that curcumin had increased apoptosis on p53 mAPK line, which was an ovarian cancer model (26).

In our study, the Tunel Staining Method was performed on solid tumours. When the tumour control group was compared with the treatment groups, the number of the cells that underwent apoptosis in the tumour control group was 5.455, whereas the number of the cells which were tunel-positive was determined to be 6.575 in the group administered with 25 mg/kg curcumin, and 7.775 in the group administered with 50mg/kg curcumin. 

Whether or not the agents investigated during the experimental studies have any anti-angiogenic effect on tumour vascularization is of importance in determining the anti-carcinogenic effectiveness of the agent Experimental studies are increasing day by day and these studies are carried out on various experimental models (26-28). In their study, El Azab *et al*. (29) examined the anti-angiogenic effects of curcumin and resveratrol in the mice to which EAT cell was injected; and stated that these two agents had suppressed microvascularization. In our study, the vascular density was evaluated by examining the expression of FVIII in solid tumour masses, and while the mean vascular density in the tumour control group was calculated as 6.033, it was calculated as 3.000 in the treatment group administered with 25mg/kg curcumin, and as 1.866 in the group administered with 50mg/kg curcumin. The decrease in FVIII expression in the treatment groups was found to be statistically significant (*P*<0.05).

Abuelba *et al*. (30) reported that 15μM-curcumin on MDA-MB-231 cell line had suppressed the tumour cell development and that curcumin had increased apoptosis as well. In the literature, it was ascertained in the *in vitro* studies conducted on various cell lines that 5-100 μM-curcumin dosages had been effective. 

## Conclusion

In our study, we performed both *in vivo* and *in vitro* examinations over the anti-tumoral effect of curcumin on EAT cells, and we determined the fact that in our *in vivo* experimental groups that high dosage treatment was influential in groups which received ascites tumour. In ascites tumour group administered with 25mg/kg curcumin; however, more EAT cells were observed in the connective tissue capsule when compared with the other treatment (50mg/kg curcumin) group.Tumour volume was measured as 4603.99mm^3^ in the tumour control group, 1179.56mm^3^ in the treatment group administered with 25mg/kg curcumin, and as 2059.12 mm^3^ in the other treatment group administered with 50mg/kg curcumin. Tumour control group was compared with the treatment groups, 50mg/kg curcumin was found to be statistically significant (*P*<0.05) in the Tunel Staining Method groups. In our study, it was seen during the counting processes performed on EAT cell line after 3 and 24-hr-incubation periods that the mean number of the dead cells in the treatment groups administered with a lower dose of curcumin (10µg/ml) had proved to be higher than that of the control group, and this difference was determined to be statistically significant (*P*<0.05).

We consider that our study will be a reference in future studies that will be conducted on curcumin in calculating the dosage. 
